# Temporal Anomaly Detection in Attention-Deficit/Hyperactivity Disorder Using Recurrent Neural Networks

**DOI:** 10.7759/cureus.76496

**Published:** 2024-12-27

**Authors:** Georgios Bouchouras, Georgios Sofianidis, Konstantinos Kotis

**Affiliations:** 1 Rehabilitation, School of Health Sciences, Metropolitan College, Thessaloniki, GRC; 2 Cultural Technology and Communication, Intelligent Systems Lab, University of the Aegean, Mytilene, GRC

**Keywords:** attention-deficit/hyperactivity disorder (adhd), intrnet of things (iot), isolation forest, machine learning, motor activity patterns, recurrent neural networks (rnn), temporal anomaly detection, wearable sensors

## Abstract

Attention-deficit/hyperactivity disorder (ADHD) is a prevalent neurodevelopmental condition marked by movement hyperactivity, often persisting into adulthood. Understanding the movement patterns associated with ADHD is crucial for improving diagnostic precision and tailoring interventions. This study leverages the HYPERAKTIV dataset, which includes high-resolution temporal data on motor activity from people diagnosed with ADHD. We used the isolation forest algorithm to detect anomalies in activity data, followed by the development of a recurrent neural network (RNN) model to predict these anomalies over time. The RNN model demonstrated high predictive accuracy, with a mean accuracy of 0.953 and a mean loss of 0.124 for participants with ADHD. These findings suggest that machine learning techniques, particularly RNNs, can effectively identify and predict anomalies in temporal motor activity data, offering objective insights into ADHD-related movement behaviors. This approach is promising for informing personalized interventions and improving clinical decision-making in the management of ADHD.

## Introduction

Attention-deficit/hyperactivity disorder (ADHD) is a prevalent neurodevelopmental disorder that manifests through symptoms of inattention, hyperactivity, and impulsivity, which can persist into adulthood [1-3]. Understanding movement patterns in individuals with ADHD is critical to improving diagnostic accuracy, intervention strategies, and overall quality of life of affected individuals. Recent advances in wearable sensor technology and activity tracking have revolutionized the ability to capture and analyze motor activity data, offering objective insights into the behavioral characteristics of ADHD [4,5]. Among these developments, the HYPERAKTIV dataset [6] stands out for its high-resolution temporal data on motor activity and physiological signals, offering a valuable resource for researchers who want to gain a deeper and more comprehensive understanding of the movements related to ADHD. Detecting anomalies within these data, defined as deviations from expected patterns, is critical to advance understanding, diagnosis, and treatment of this neurodevelopmental condition [7].

By identifying anomalies in movement behaviors, researchers can uncover new insights into ADHD, paving the way for improved intervention strategies and improved quality of life for affected individuals. Advanced computational models, such as recurrent neural networks (RNNs), offer the ability to analyze temporal activity data and detect patterns that might serve as objective markers of ADHD-related behaviors [8]. Although these markers can complement traditional diagnostic tools, their role in enabling continuous monitoring of treatment efficacy, personalizing interventions, and providing a deeper understanding of movement patterns remains an area requiring further exploration and validation [9].

Traditional studies have primarily focused on statistical summaries or basic feature extraction from behavioral data, leaving a gap in research that uses advanced machine learning (ML) techniques for temporal anomaly detection [10].

This study aims to fill this gap by employing the isolation forest algorithm [11], which is effective for anomaly detection in high-dimensional datasets, and developing an RNN model [12] to predict these anomalies over time. RNNs are particularly suitable for this task due to their ability to capture temporal dependencies in sequential data, making them ideal for analyzing the time series nature of motor activity data in people with ADHD [7].

The primary research question that guides this study is whether ML techniques can effectively detect and predict anomalies in temporal motor activity data from people with ADHD. By identifying anomalies, this research seeks to uncover behavioral patterns that may be unique to ADHD participants, potentially forming personalized interventions and improving clinical decision-making. The implications of this research extend beyond academic inquiry, as understanding these behavioral patterns can lead to better therapeutic strategies and better outcomes for individuals with ADHD [4,5]. For instance, wearable sensor technology can continuously monitor motor activity, detecting anomalies that may signal treatment inefficacy or the need for intervention adjustments. By analyzing these real-time data streams, clinicians can gain actionable insights into how well a treatment manages ADHD-related motor anomalies. This could allow for timely modifications of therapy, improving its effectiveness and tailoring interventions to the individual’s needs, ultimately contributing to more personalized and dynamic care strategies.

Related work

Machine Learning/Deep Learning and ADHD

Recent studies have highlighted the potential of using machine learning (ML and deep learning techniques for the automatic assessment of ADHD-related anomalies. For example, Munoz-Organero et al. developed a convolutional neural network to analyze movement patterns in children with ADHD, utilizing data from accelerometers to capture characteristic motor behaviors that differ from those of neurotypical children [13]. This approach exemplifies how ML can facilitate the identification of subtle behavioral anomalies that are often overlooked in traditional assessments. Moreover, Chatterjee et al. approached the problem from a network analysis perspective, employing the Forman-Ricci curvature to detect changes in the functional connectivity of brain networks in ADHD patients. Their findings suggest that ADHD is associated with significant structural changes in brain networks, which can be framed as a network anomaly detection problem [14]. This innovative methodology underscores the importance of understanding ADHD not just as a behavioral disorder but as one that involves profound neurological alterations. In addition to behavioral and network analyses, neuroimaging studies have played a crucial role in identifying structural anomalies in individuals with ADHD. Liu et al. reported alterations in brain structural networks, particularly in cortical thickness, which are indicative of the neurodevelopmental aspects of ADHD [15]. These findings align with the work of Shaw et al., who conducted a longitudinal study on cerebellar development in ADHD, revealing that atypical cerebellar growth may contribute to the disorder's symptoms [16]. Such neuroanatomical insights are vital for developing targeted interventions and improving diagnostic accuracy. Furthermore, the integration of genetic studies into the anomaly detection framework has provided a deeper understanding of ADHD's etiology. Thapar's review of genetic discoveries related to ADHD emphasized the significant genetic overlaps with other neurodevelopmental disorders, which could inform the identification of biomarkers for ADHD [17]. This genetic perspective can improve the precision of anomaly detection by correlating specific genetic markers with observed behavioral and neuroimaging anomalies. Finally, the role of environmental factors, such as maternal influences and childhood trauma, has been acknowledged in the context of ADHD. Banik et al. discussed how maternal factors can induce epigenetic changes that contribute to neurological disorders, including ADHD [18]. This highlights the multifaceted nature of ADHD, where both genetic predispositions and environmental triggers can lead to observable anomalies.

Wearables, Internet of Things, and ADHD

The intersection of wearable technology, the internet of things (IoT), and ADHD has garnered increasing attention in recent years, particularly as digital health interventions (DHIs) emerge as promising avenues for managing this prevalent neurodevelopmental disorder. The IoT refers to a network of physical devices, such as wearable sensors, smartphones, and home appliances, that are connected to the internet and can collect, share, and analyze data automatically.

Wearable technologies have been identified as valuable tools in the management of ADHD, particularly through their ability to monitor physiological and behavioral data in real time. For instance, Wu et al. discussed how emerging wearable biosensors can be integrated with applications to enhance cognitive function and attention, which are critical areas of concern for individuals with ADHD [19].

Similarly, the study by Leikauf et al. presented an Apple Watch application designed to track movement and provide feedback to users, demonstrating the feasibility of using wearables to support self-monitoring in ADHD management [20]. These devices can facilitate non-pharmacological interventions, which are increasingly recognized as essential for comprehensive ADHD treatment strategies [21]. Moreover, the integration of IoT with wearable devices allows for continuous data collection and analysis, enabling personalized interventions.

The CareWear project exemplifies this by developing an online platform that utilizes data from wearables to inform mental health care practices. Such platforms can enhance the clinical utility of wearables by providing healthcare professionals with actionable insights derived from real-time data, thereby improving treatment outcomes for ADHD patients [22].

Additionally, the use of mobile health (mHealth) technologies, as discussed by Schoenfelder et al., has shown promise in increasing physical activity among adolescents with ADHD, which, in turn, can mitigate symptoms [23].

Despite the potential benefits, several challenges remain in the implementation of wearable technologies for ADHD management. Issues such as data accuracy, user engagement, and the need for robust validation of wearable applications are critical [19]. Furthermore, the effectiveness of these technologies in real-world settings requires further investigation, as highlighted by Hollis et al., who call for more research on the sustainability and generalization of DHIs beyond controlled environments The need for human facilitation and reminders also plays a significant role in the success of these interventions, emphasizing the importance of integrating behavioral support alongside technological solutions [21].

In conclusion, the convergence of wearables, IoT, and ADHD management presents a promising frontier in mental health care. While current research underscores the potential of these technologies to enhance treatment and monitoring, further studies are necessary to address existing challenges and validate their efficacy in diverse populations. Future research should focus on developing scalable, user-friendly solutions that can be seamlessly integrated into daily life, thereby improving the overall quality of care for individuals with ADHD. In this paper, authors do not utilize an approach for collecting and managing ADHD-related data, but they utilize an existing open dataset for the purpose of experimenting with the automatic assessment of ADHD-related anomalies.

## Materials and methods

Data source

The activity data used in this study originate from the HYPERAKTIV dataset [6] (dataset available at https://osf.io/3agwr), a publicly available resource designed for research on ADHD and related mental health disorders. This data set comprises sensory data, including measurements of motor activity and heart rate, collected from patients diagnosed with ADHD and clinical controls. The dataset contains activity recordings segmented into individual files for each participant, detailing time-stamped activity levels. The activity data used in the HYPERAKTIV dataset were collected using a wrist-worn actigraph device (Actiwatch, model AW4, Cambridge Neurotechnology Ltd, Eastleigh, England) equipped with a piezoelectric accelerometer. This device recorded three-dimensional movement data (x, y, z axes) with a sampling frequency of 32 Hz. Movements exceeding 0.05 g (instances where the acceleration recorded by the device surpassed a threshold of 0.05 times the force of gravity (g)), which is equivalent to detecting small but noticeable physical activities, were recorded and aggregated into 1-minute epochs, generating an integer value proportional to the intensity of the movement. Therefore, in general, these values represent a processed magnitude of acceleration derived from readings from the three-axis accelerometer. Data collection spanned an average of 6.6 ± 1.3 days for 51 ADHD participants. These high-resolution temporal activity data served as the basis for analyzing movement patterns and detecting anomalies associated with ADHD. Controls were not taken into account in this study, as our primary objective was to focus on identifying anomalies specifically in people with ADHD.

Anomaly detection

To analyze the temporal patterns of activity, we first applied an anomaly detection algorithm to each participant’s activity data using the isolation forest technique. The steps were as follows:

(1) Data loading: each participant’s activity data, stored as.csv files, was loaded and pre-processed. Each file included:

 • TIMESTAMP: The time of the recorded activity.

 • ACTIVITY: A numerical value indicating the activity value.

(2) Algorithm setup: an isolation forest model was trained on the ACTIVITY column for each file to identify anomalies. A contamination parameter of 0.05 was used, which means that 5% of the data was assumed to be anomalous [7]. The model was also configured with a random state of 42 to ensure reproducibility.

(3) Anomaly labeling: the model output classified each data point as normal (0) or anomaly (1). This classification was added as a new column, ANOMALY, in the processed files.

(4) Result export: the processed files with anomaly labels were saved for downstream analysis.

**Figure 1:**
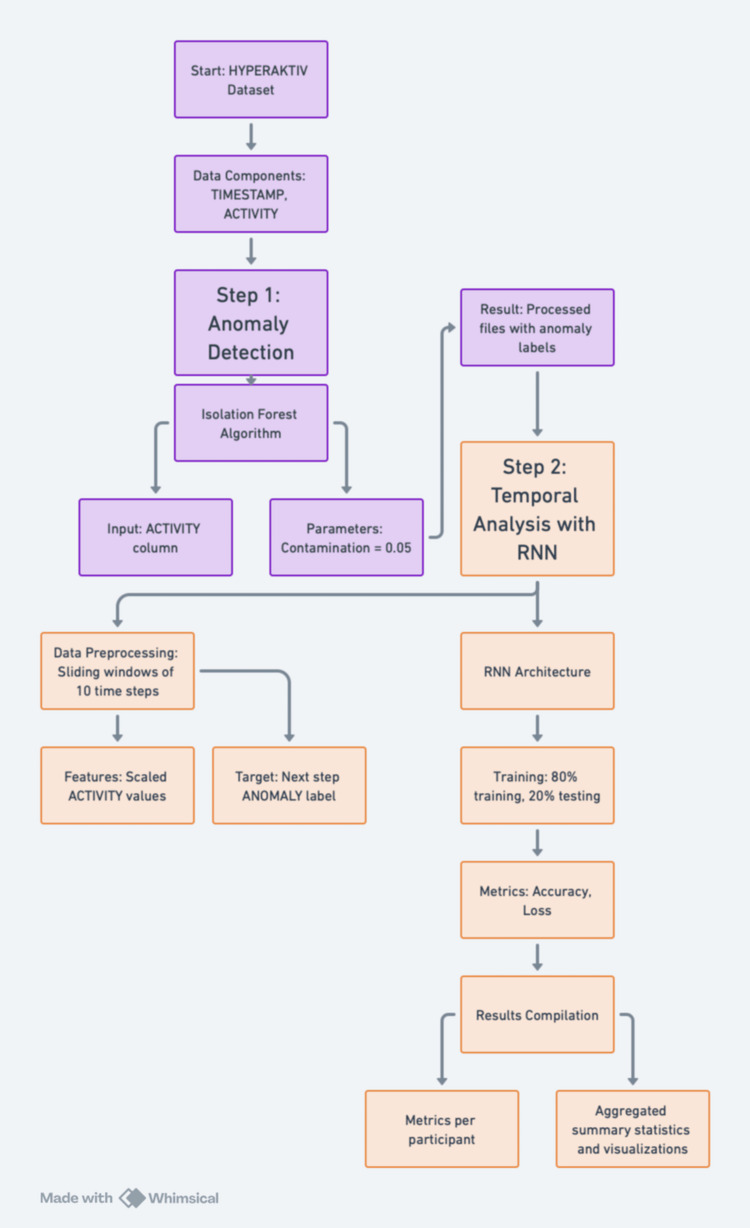
Flowchart of the methodology used in this study. https://whimsical.com

The steps are also illustrated in Figure 1.

RNN implementation and workflow

To analyze temporal patterns in anomalies, we utilized an RNN model built with Tensor-Flow/Keras. The implementation followed these steps:

Step 1: Input Data

Normalized activity data was divided into sequences of 10 time steps:

$x = [x_1, x_2, \ldots, x_{10}]$

where x_t_ represents the activity value at time t

Step 2: Hidden State Calculation

At each time step, the hidden state (ht) was updated using:

$h_t = \phi(W_{xh}x_t + W_{hh}h_{t-1} + b_h)$

where W_xh_ are the input-to-hidden weights, W_hh_ are the hidden-to-hidden weights, b_h_ is the bias term, and ϕ is the activation function (ReLU).

Step 3: Output Prediction

After processing 10 time steps, the final output was derived from the last hidden state:

$y = \sigma(W_{hy} h_{10} + b_y)$

where W_hy _are the hidden-to-output weights, b_y_ is the bias term, and σ is the sigmoid activation function for anomaly probability.

Step 4: Loss Function

Binary cross-entropy was used to measure prediction accuracy:

$\text{Loss} = -\frac{1}{N} \sum_{i=1}^{N} \left[ y_i \log(\hat{y}_i) + (1 - y_i) \log(1 - \hat{y}_i) \right]$

where N is the number of samples, y_i_ is the true label, and ˆy_i_ is the predicted probability.

Step 5: Training

 • Weights (W_xh_, W_hh_, W_hy_) were optimized using the Adam algorithm.

 • Early stopping was employed over 20 epochs to prevent overfitting.

Details of the RNN design are as follows:

(1) Data preprocessing: time-series windows of 10 time steps were created. The ANOMALY

column was the target variable (0: normal, 1: anomalous). Data was split into 80% training and 20% testing.

(2) Model architecture: the RNN consisted of one SimpleRNN layer with 50 units and

ReLU activation, followed by a dense output layer with sigmoid activation.

(3) Evaluation: metrics (accuracy, loss) were calculated for the test data, and results were aggregated into a summary CSV.

More details on the custom codes used for data preprocessing, anomaly detection, and RNN analysis, along with the complete implementation, can be found in the GitHub repository linked to this study (https://github.dev/GiorgosBouh/adhd_anomaly_detection_RNN). The repository provides step-by-step instructions and all required scripts to reproduce the results presented in this study.

## Results

The analysis of model performance revealed insightful findings regarding the RNN model's ability to classify anomaly detection based on temporal activity data. For the ADHD participants, the RNN model achieved the summary statistics seen in Table 1.

**Table 1:** Summary of descriptive statistics for model performance.

Metric	Mean	Standard Deviation
Accuracy	0.953	0.021
Loss	0.124	0.044

**Figure 2:**
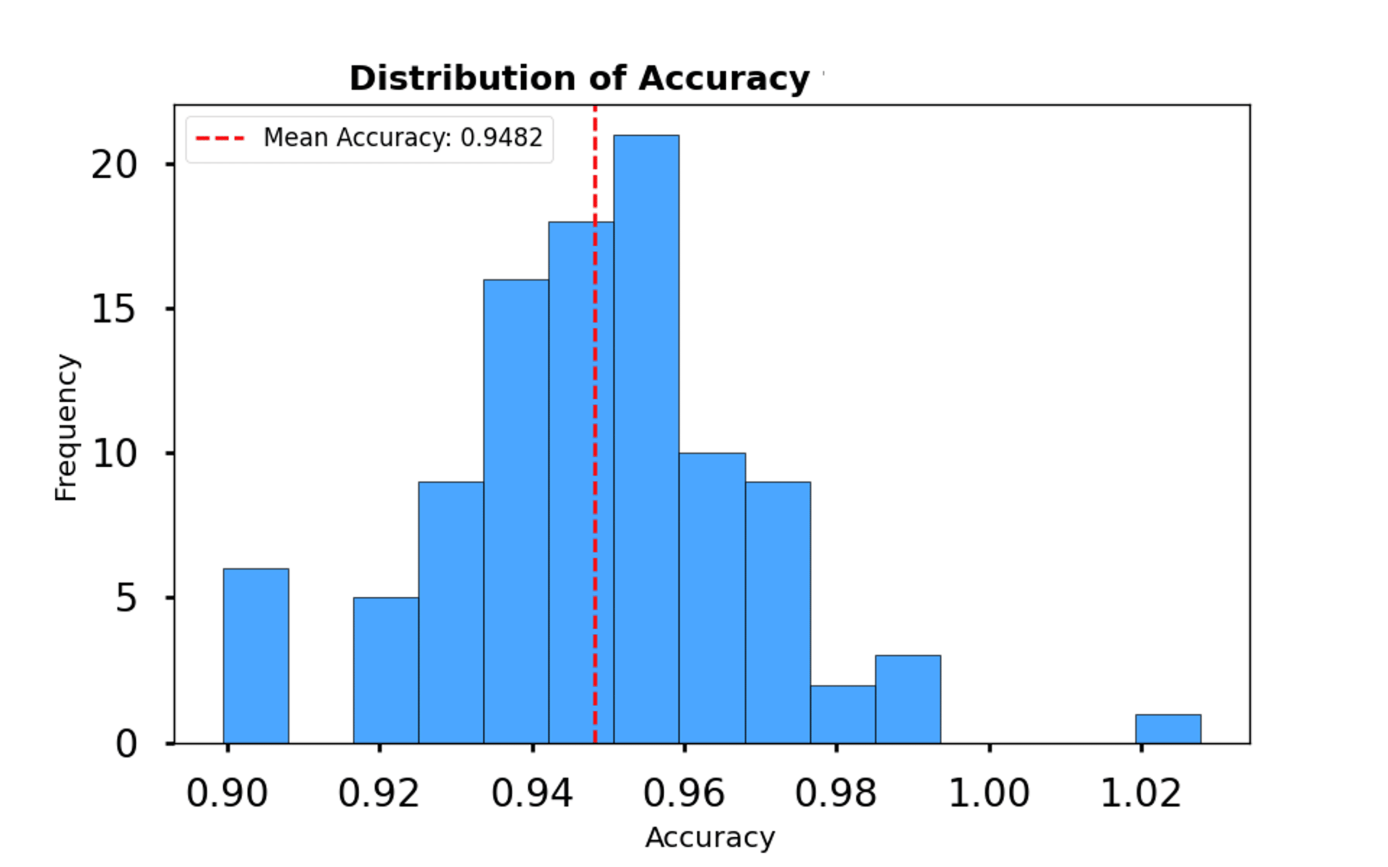
Distribution of accuracy for participants with attention-deficit/hyperactivity disorder. The red dashed line represents the mean accuracy of 0.95.

**Figure 3:**
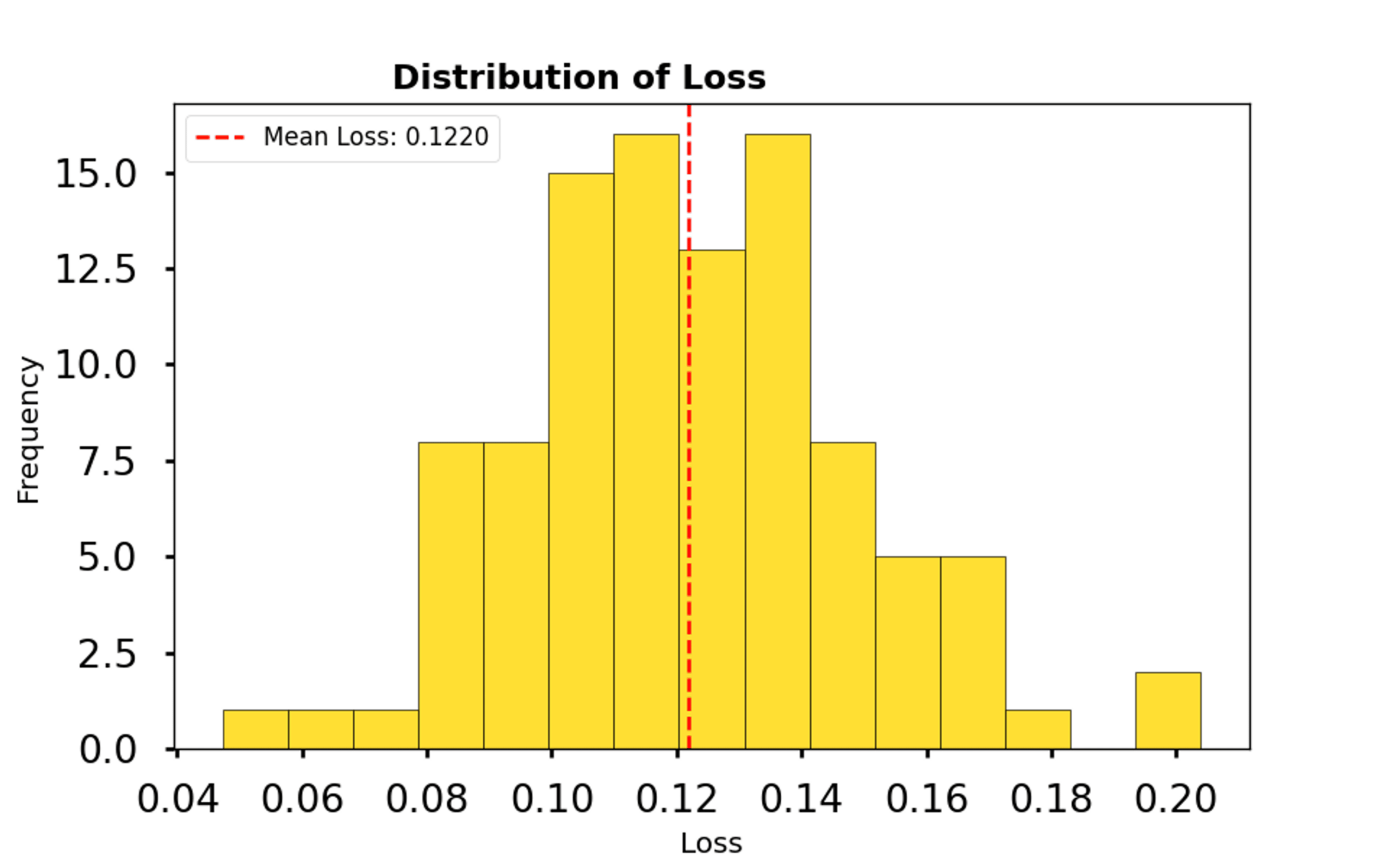
Distribution of loss for participants with attention-deficit/hyperactivity disorder. The red dashed line represents the mean loss of 0.124.

The accuracy distribution is shown in Figure 2. The histogram reveals that most participants achieved high accuracy values, with a mean of 0.953. This reflects the model's consistent ability to correctly classify anomalies in the activity data. The loss distribution is shown in Figure 3. The histogram illustrates that most participants had low loss values, with a mean loss of 0.124. This low error further highlights the model's ability to minimize prediction deviations and achieve robust performance.

The high mean accuracy (0.953) and low mean loss (0.124) suggest that the RNN model performs reliably for the participants. The low standard deviation for both metrics indicates consistency across people with ADHD, further supporting the model's reliability.

The findings demonstrate the potential of the model to accurately detect anomalies in temporal activity data, providing a robust approach to analyze behavioral patterns in participants with ADHD.

## Discussion

The results of this study demonstrate the potential of ML models to analyze temporal activity data in individuals with ADHD. The high mean accuracy (0.953) and low mean loss (0.124) achieved by the RNN model in anomaly classification for participants with ADHD underscore the model's effectiveness in capturing temporal patterns and distinguishing anomalous behaviors from typical activity. These findings are significant for a variety of reasons.

Traditional approaches to analyzing ADHD often rely on subjective assessments or cross-sectional data, which may not fully capture the dynamic nature of behavior [24]. Using temporal activity data, as this study did, provides a more granular understanding of behavioral patterns, paving the way for objective and data-driven evaluations [8]. The ability to analyze data over time allows for a more detailed view of how ADHD symptoms manifest in daily life, which is crucial for effective intervention [8,25]. For instance, Munoz-Organero et al. utilized CNNs to analyze ADHD-related movement patterns and demonstrated the potential of ML for detecting subtle behaviors often missed by traditional assessments [13]. Similarly, Chatterjee et al. used network analysis to identify brain connectivity anomalies in ADHD patients, showcasing the utility of advanced computational models in understanding ADHD behaviors [14]. The current study findings align with these studies by further validating the use of ML, specifically RNNs, for analyzing time-series data to detect behavioral anomalies objectively. Using temporal activity data, as this study did, provides a more granular understanding of behavioral patterns, paving the way for data-driven evaluations. This method complements prior work by Liu et al., who identified structural brain anomalies in ADHD patients, and demonstrates how temporal motor activity data can add value to understanding dynamic behavior [15]. The ability to analyze data over time allows for a detailed view of how ADHD symptoms manifest daily, which is crucial for effective intervention.

The ability to accurately detect anomalies in motor activity data could serve as a valuable tool to monitor ADHD symptoms and evaluate treatment responses. For example, periods of abnormal activity can correspond to moments of increased hyperactivity, inattention, or other clinically relevant behaviors [26]. This capability could improve clinical decision-making by providing real-time insight into patient behavior, thus informing personalized treatment strategies [27]. The low standard deviation in accuracy and loss metrics suggests that the RNN model is reliable among participants with ADHD. This consistency underscores the generalizability of the approach within this population, supporting its potential application in broader clinical and research settings. The robustness of the model is particularly important in clinical contexts, where variability in patient responses can complicate treatment [28]. The use of RNNs allowed analysis of temporal dependencies in activity data, capturing trends and patterns that might be missed by static models. This methodological innovation represents a step forward in using ML for behavioral research, as it allows the identification of changes in behavior over time by analyzing movement patterns, which are closely related to the progression of symptoms of ADHD, such as hyperactivity [29].

Despite these promising findings, there are limitations to consider. The dataset used in this study, while comprehensive, is limited to mostly adults with ADHD and may not fully represent the heterogeneity of the ADHD population. Furthermore, while the RNN model performed well, its reliance on past data to predict anomalies raises questions about its interpretability and application in real-time scenarios. Future work should explore ensemble methods and hybrid models to improve predictive power and interpretability. In conclusion, this study demonstrates the feasibility and utility of using ML models, particularly RNNs, to detect and analyze anomalies in temporal motor activity data from adults with ADHD. These results contribute to the growing body of evidence supporting the integration of ML in clinical research and practice, providing a foundation on which future studies can build.

## Conclusions

The study highlights the effectiveness of RNNs in analyzing temporal activity data for individuals with ADHD in detecting anomalies, which enhances the understanding of ADHD-related behaviors. This capability to accurately identify anomalies in motor activity offers valuable insights for monitoring ADHD symptoms and evaluating treatment responses, ultimately improving clinical decision-making and enabling personalized treatment strategies.

## References

[1] Anderson A, Douglas PK, Kerr WT (2014). Non-negative matrix factorization of multimodal MRI, fMRI and phenotypic data reveals differential changes in default mode subnetworks in ADHD. Neuroimage.

[2] Monastra VJ, Lubar JF, Linden M (1999). Assessing attention deficit hyperactivity disorder via quantitative electroencephalography: an initial validation study. Neuropsychology.

[3] Morkem R, Patten S, Queenan J, Barber D (2020). Recent Trends in the prescribing of ADHD medications in canadian primary care. J Atten Disord.

[4] Pawaskar M, Fridman M, Grebla R, Madhoo M (2020). Comparison of quality of life, productivity, functioning and self-esteem in adults diagnosed with ADHD and with symptomatic ADHD. J Atten Disord.

[5] Ludyga S, Mücke M, Leuenberger R (2022). Behavioral and neurocognitive effects of judo training on working memory capacity in children with ADHD: a randomized controlled trial. Neuroimage Clin.

[6] Hicks SA, Stautland A, Fasmer OB (2021). HYPERAKTIV: an activity dataset from adult patients with attention-deficit/hyperactivity disorder (ADHD). MMSys '21: Proceedings of the 12th ACM Multimedia Systems Conference.

[7] Aminikhanghahi S, Cook DJ (2017). A survey of methods for time series change point detection. Knowl Inf Syst.

[8] Kautzky A, Vanicek T, Philippe C (2020). Machine learning classification of ADHD and HC by multimodal serotonergic data. Transl Psychiatry.

[9] Sáez-López Sáez-López, José Manuel Buceta-Otero, D. Rogelio (2023). The M Bot robot for learning Cartesian coordinates in secondary education. Pixel-Bit, Revista de Medios y Educacion.

[10] Bulut O, Gorgun G, He S (2024). Unsupervised anomaly detection in sequential process data insights from PIAAC problem-solving tasks. J Psychol.

[11] Liu FT, Ting KM, Zhou Z-H (2008). Isolation forest. Eighth IEEE International Conference on Data Mining.

[12] Salem FM (2022). Recurrent Neural Networks: From Simple to Gated Architectures. Springer.

[13] Muñoz-Organero M, Powell L, Heller B, Harpin V, Parker J (2018). Automatic extraction and detection of characteristic movement patterns in children with ADHD based on a convolutional neural network (CNN) and acceleration images. Sensors (Basel).

[14] Chatterjee T, Albert R, Thapliyal S, Azarhooshang N, DasGupta B (2021). Detecting network anomalies using Forman-Ricci curvature and a case study for human brain networks. Sci Rep.

[15] Liu T, Chen Y, Li C, Li Y, Wang J (2017). Altered brain structural networks in attention deficit/hyperactivity disorder children revealed by cortical thickness. Oncotarget.

[16] Shaw P, Ishii-Takahashi A, Park MT (2018). A multicohort, longitudinal study of cerebellar development in attention deficit hyperactivity disorder. J Child Psychol Psychiatry.

[17] Thapar A (2018). Discoveries on the genetics of ADHD in the 21st century: new findings and their implications. Am J Psychiatry.

[18] Banik A, Kandilya D, Ramya S, Stünkel W, Chong YS, Dheen ST (2017). Maternal factors that induce epigenetic changes contribute to neurological disorders in offspring. Genes (Basel).

[19] Wu J, Li P, Luo H, Lu Y (2022). Complementary and alternative medicine use by ADHD patients: a systematic review. J Atten Disord.

[20] Leikauf JE, Correa C, Bueno AN, Sempere VP, Williams LM (2021). StopWatch: pilot study for an Apple Watch application for youth with ADHD. Digit Health.

[21] Hollis C, Falconer CJ, Martin JL, Whittington C, Stockton S, Glazebrook C, Davies EB (2017). Annual Research Review: digital health interventions for children and young people with mental health problems - a systematic and meta-review. J Child Psychol Psychiatry.

[22] Debard G, De Witte N, Sels R, Mertens M, Van Daele T, Bonroy B (2020). Making wearable technology available for mental healthcare through an online platform with stress detection algorithms: the CareWear project. J Sens.

[23] Schoenfelder E, Moreno M, Wilner M, Whitlock KB, Mendoza JA (2017). Piloting a mobile health intervention to increase physical activity for adolescents with ADHD. Prev Med Rep.

[24] Brook JS, Brook DW, Zhang C, Seltzer N, Finch SJ (2013). Adolescent ADHD and adult physical and mental health, work performance, and financial stress. Pediatrics.

[25] Agnew-Blais JC, Polanczyk GV, Danese A, Wertz J, Moffitt TE, Arseneault L (2016). Evaluation of the persistence, remission, and emergence of attention-deficit/hyperactivity disorder in young adulthood. JAMA Psychiatry.

[26] Lis S, Baer N, Stein-en-Nosse C, Gallhofer B, Sammer G, Kirsch P (2010). Objective measurement of motor activity during cognitive performance in adults with attention-deficit/hyperactivity disorder. Acta Psychiatr Scand.

[27] Langmaid RA, Papadopoulos N, Johnson BP, Phillips J, Rinehart NJ (2016). Movement scaling in children with ADHD-combined type. J Atten Disord.

[28] Monden Y, Dan I, Nagashima M (2015). Individual classification of ADHD children by right prefrontal hemodynamic responses during a go/no-go task as assessed by fNIRS. Neuroimage Clin.

[29] Konrad K, Neufang S, Fink GR, Herpertz-Dahlmann B (2007). Long-term effects of methylphenidate on neural networks associated with executive attention in children with ADHD: results from a longitudinal functional MRI study. J Am Acad Child Adolesc Psychiatry.

